# Variations of pulmonary arteries and other associated defects in Tetralogy of Fallot

**DOI:** 10.1186/2193-1801-3-467

**Published:** 2014-08-26

**Authors:** Abdul Malik Sheikh, Uzma Kazmi, Najam Hyder Syed

**Affiliations:** Department of Paediatric Cardiology, The Children Hospital and The Institute of Child Health, Ferozpur Road, Lahore, Punjab, Pakistan

**Keywords:** *Cardiac catheterization*, *Tetralogy of Fallot*, *Pulmonary artery variants*

## Abstract

**Background:**

The objective of study was to determine pulmonary artery variations and other associated cardiac defects in patients with Tetralogy of Fallot. This cross-sectional, descriptive study was carried out at The Children's Hospital and the Institute of Child Health, Lahore, from January 2006 to December 2012. All patients with Tetralogy of Fallot, who underwent cardiac catheterization during this period, were included. Standard cine-angiograms were done to record the pulmonary artery sizes and associated cardiac defects.

**Description:**

A total of 576 patients with Tetralogy of Fallot were catheterized. Pulmonary Artery abnormalities were present in 109 (18.92%) patients. The commonest abnormality was isolated Left Pulmonary Artery stenosis (n = 60, 10.4%) followed by supra-valvular stenosis (n = 9, 1.6%). Left Pulmonary Artery was absent in seven patients(1.2%), while 1 patient (0.2%) had both absent right and left Pulmonary Arteries with segmental branch pulmonary arteries originating directly from Main Pulmonary Artery. Associated cardiac lesions included right aortic arch in 72 (12.5%), additional muscular Ventricular Septal Defect in 31 (5.4%), Patent Ductus Arteriosus in 31 (5.4%), bilateral Superior Vena Cava 36(6.2%), Atrial Septal Defect 4(0.7%) and Major Aortopulmonary Collateral Arteries in 75(13%) patients. Significant coronary artery abnormalities were present in 28(4.9%) children.

**Conclusion:**

Pulmonary artery abnormalities were present in 18.92% of patients with Tetralogy of Fallot. Isolated Left Pulmonary Artery origin stenosis was the most common abnormality. Significant associated cardiac lesions including Patent Ductus Arteriosus , additional muscular Ventricular Septal Defect, coronary artery abnormalities, bilateral Superior Vena Cava, Atrial Septal Defect and Major Aortopulmonary Collateral Arteries were present in one-third of the patients.

## Background

Tetralogy of Fallot (TOF) is the most common cyanotic congenital heart disease; with an incidence of approximately 0.34/1000 live births making 5-7% of congenital heart lesions. TOF is a congenital cardiac anomaly characterized by a large ventricular septal defect (VSD), muscular obstruction within the right ventricular outflow tract, overriding of aorta and the right ventricular hypertrophy (van der Linde et al. 2011; Ho et al. 2007; Elzenga et al. 1990). In the current era, TOF is almost universally amenable to surgical repair with good long-term outcome. This, however, requires a thorough pre-operative anatomic description of central and branch pulmonary arteries and associated defects, like additional muscular VSD, ductus arteriosus (PDA), Major Aortopulmonary Collateral Arteries (MAPCA) for better surgical planning and a better outcome (Mølgaard-Nielsen et al. 2013; Dodge-Khatami 2014). Echocardiography with Doppler interrogation gives an accurate diagnosis of anatomy of these patients. Angiography, however, compliments the echocardiographic study as it allows more accurate evaluation of pulmonary vasculature, coronary arteries and additional ventricular septal defects (Aboulhosn 2013; Mishra et al. 2014; Kasar et al. 2011). There is a limited data on these variations and associations in the local population where patients usually present late in more severe form. This study was designed to determine various anatomic variations in pulmonary vasculature and other associated cardiac defects in patients with TOF.

## Methodology

This cross-sectional descriptive study was conducted at the Children's Hospital and the Institute of Child Health, Lahore, from January 2006 to December 2012. The patients with echocardiographic diagnosis of TOF, undergoing cardiac catheterization during the study period, were included. Patients with Complete Atrioventricular Septal Defect with TOF, pulmonary atresia with VSD and operated cases of BlalockTaussig shunt for TOF were excluded. Approval of the hospital's Ethics Committee was obtained for the study and parents of patients gave informed consent. A majority of patients underwent cardiac catheterization under local anesthesia and sedation. A cocktail of 1% morphine (0.1 mg/kg) with chlorpromazine and an antiemetic was given half an hour before procedure to all patients. Effective sedation and analgesia were maintained during the procedure, using midazolam and ketamine^7^. Right and left cardiac catheterizations were carried out. Pressures were recorded and oximetry was carried out in the standard manner. Cine-angiograms were done in the recommended positions. Pulmonary artery sizing was done using z-scoring, Mcgoon ratio and Nakata index. A value of < -3 was taken to describe hypoplasia^8^. Data were analyzed using SPSS version 19. The variables like age and gender were presented as simple descriptive statistics; calculating mean and standard deviation of numerical data (age) and frequency percentage for qualitative data (gender). The percentages of outcome variables like associated lesions and anatomic variations were calculated. Since, this was a descriptive diagnostic study; no test of significance was applied.

## Description

A total of 576 patients with TOF underwent cardiac catheterization during the study period. There were 378 (65.6%) males and 198 (34.4%) females. The age of presentation was 6 months to 18 years with a mean of 69 (+43.14) months. Majority (52.3%) were between 6 months and 5 years of age, followed by 35.8% patients between 5–10 years and 11.9% between 10–18 years.

All patients had levocardia except 2 patients with dextrocardia. Five hundred and sixty nine patients had situs solitus while 1 had situs inversus and 6 with situs ambiguous. Pulmonary artery variations were detected in 109 (18.92%) patients. Regarding anatomic variations of pulmonary artery and its branches, Left pulmonary artery(LPA) stenosis was detected as the most common lesion present in 60 (10.4%) patients followed by supravalvular stenosis in 9 (1.6%). The frequency of various pulmonary artery abnormalities is given in Table 1. Of patients with LPA stenosis; 7 had associated Patent ductus arteriosus (PDA) as well. Fifty three cases of PDA were found in those cases where there was no pulmonary artery stenosis. The patients having LPA and RPA stenosis and absent LPA also had PDA.

**Table 1:** **Pulmonary artery abnormalities found in catheterized patients of Tetralogy of Fallot (n = 138)**

Pulmonary artery abnormality	Frequency (percentage)
Isolated LPA stenosis	60(10.4)
Isolated RPA stenosis	5(0.9)
MPA and LPA origin stenosis	4(0.7)
RPA and LPA stenosis	4(0.7)
Supravalvular stenosis	9(1.6)
Isolated MPA hypoplasia	4(0.7)
Isolated LPA hypoplasia	6(1)
Isolated RPA hypoplasia	1(0.2)
Uniform pulmonary artery hypoplasia	3(0.5)
Hypoplastic MPA and LPA	1(0.2)
Absent LPA , arising from Aorta	7(1.2)
Absent LPA and RPA	1(0.2)
RPA hypoplasia with LPA dilatation	1(0.2)
MPA, LPA and RPA stenosis	2(0.3)
MPA and LPA origin stenosis, distal PA stenosis	1(0.2)
Absent RPA	1(0.2)
Total	109(18.9)

Associated cardiac lesions found in catheterized patients of TOF are shown in Table 2. All patients had normal origin of head and neck vessels. Additional muscular VSD was single in 25 (4.3%) patients and multiple in 5 (0.9%) patients. Fourteen (2.4%) patients had prominent conal branch of right coronary artery crossing Right Ventricular Outflow Tract (RVOT), while 14 (2.4%) patients had common origin of coronary artery from left coronary cusp. Bilateral Superior Vena Cava (SVC) was found in 36 (6.2%) patients.

**Table 2:** **Associated cardiac lesions found in catheterized patients of tetralogy of Fallot**

Associated cardiac anatomic defects	Frequency (percentage)
Right aortic arch	72 (12.5)
PDA	31(5.4)
Additional VSD	31 (5.4)
Coronary artery abnormalities	28 (4.9)
Bilateral SVC	36 (6.2)
MAPCA	75 (13)
ASD	4 (0.7)

## Discussion

Tetralogy of Fallot is the commonest cyanotic congenital heart defect (Garekar & Humes 2013; Kemper et al. 2011). Surgery is the standard form of treatment and cine-angiogram has been the Gold standard in pulmonary vasculature assessment to delineate anatomy prior to surgery. Magnetic resonance angiography has replaced the cine-angiogram in the modern era (Siripornpitak et al. 2013; Garg et al. 2014). The age of presentation in this study was relatively older with majority between 6 month and 5 years of age. Farasani showed same age of presentation in Iranian population(Farsani 2007). In Europe and North America frequently the diagnosis of TOF can be made prenatally, and the current policy is to correct TOF after 4–6 months of life (Wang et al. 2007; Bailliard & Anderson 2009; Apitz et al. 2009). This disparity in Asian and European population is due to less efficient primary and secondary healthcare structure to pick-up all children born with a cyanotic CHD.

**Figure 1:**
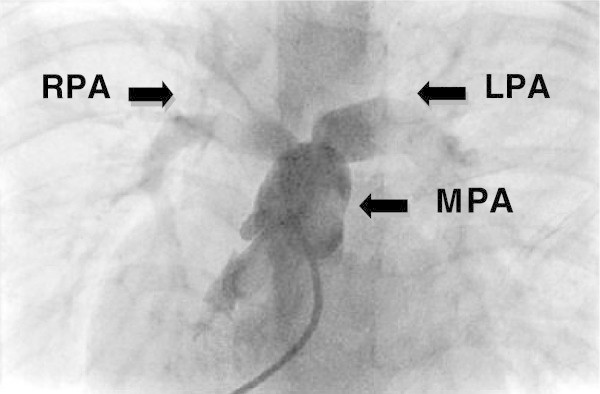
**Bilateral branch pulmonary arteries origin stenosis.** RPA: Right Pulmonary Artery; LPA: Left Pulmonary Artery; MPA: Main Pulmonary Artery.

The overall incidence of pulmonary artery abnormalities in our population was found to be 18.92%, which is comparable to the data collected by Sharma (Sharma et al. 1989). Bacha noted pulmonary artery abnormalities in 20% cases (Bacha & Kreutzer 2001). Wang recorded pulmonary artery abnormalities in 18.75% cases (Wang et al. 2007) .The incidence was recorded very high (up to 40%) in some studies (Saeed et al. 2009; Harikrishnan et al. 2000). The discrepancy may be due to large number of cases registered in our study as compare to previous studies. Isolated LPA stenosis was detected in 10.4% of this study cases. This is comparable to the reported incidence of 3% and 10% from Asia and Europe respectively_._ Stenosis of RPA (0.9%) is also comparable with data by Farasani, where it was found to be 2.2%. In this data, combined MPA and LPA origin stenosis was 0.7% again consistent with same Iranian population, where it was found to be in 0.7% (Elzenga et al. 1990; Farsani 2007) (Figure 1).

Haikrishna found that discrete stenosis was significantly present in patients with PDA 67/84 than without PDA 5/96 (Harikrishnan et al. 2000). Similar association was confirmed by other workers (Moon-Grady et al. 2007). In this study, association of PDA with pulmonary artery stenosis was found in only 7 out of 60 patients of LPA stenosis at the time of catheterization. This, however, does not exclude possible complete closure of PDA leading to LPA stenosis diagnosed on angiography subsequently. The likely cause of pulmonary artery stenosis in TOF with PDA may be the opposing flows through RVOT and PDA producing a watershed effect at the ductus-pulmonary artery junction.

Supravalvular stenosis was present in 9 (1.6%) cases, second most common pulmpnary artery variant in our study. Agrawal and colleagues showed higher percentage this abnormality (33%) (Agrawal et al. 1990). This discrepancy may be due to inclusion of elder patients (5–50 years) in their study.

In this series, coronary artery variations were in 4.9% cases which were comparable to 5-7% as noted by Gupta (Gupta et al. 2001). Conal branch crossing RVOT was found in 2.45% of TOF patients same as documented incidence of upto 4% (Topaz et al. 1992). Frasani recorded this abnormality in 15% cases(Farsani 2007). Single origin of coronary arteries was found in 2.45% cases as compared to 4–4.5% in other worker's results (Farsani 2007). Rest of the coronary artery abnormalities like left anterior descending from right coronary artery, left circumflex from right coronary artery and conal branch from left anterior descending artery were not detected in this group of patients.

Right aortic arch has no functional significance but it is important to tell surgeons beforehand to avoid complications. Right aortic arch was found in 12.5% cases as opposed to documented incidence of 20–25% cases (Kemper et al. 2011; Siripornpitak et al. 2013).However Saeed and colleagues found right arch in 15% patients, same as in our study (Saeed et al. 2009).

PDA was found in 5.4% cases same as recorded by studies by other workers (Farsani 2007; Harikrishnan et al. 2000; Rasul et al. 2008). Bilateral SVC were found in 6.2% of patients. Bilateral SVC were recorded as low as 2.8% to as high as 11% (Saeed et al. 2009). This finding is important for the cannulation prior to cardiopulmonary bypass.

Major Aortopulmonary Collateral Arteries (MAPCA) were present in 75(13%) patients. Rasul and colleagues found MAPCA in less than 5% cases (Grosse-Wortmann et al. 2013). Farsani found MAPCA in 1.1% cases (Farsani 2007). Higher percentage in our study was due to late presentation and more severe disease.

## Conclusion

The frequency of pulmonary artery abnormalities in the studied population of patients with TOF was high (18.92%). Commonest pulmonary artery abnormality was isolated LPA stenosis (10.4%) followed by supravalvular pulmonary stenosis in 1.6%. Important associated cardiac lesions included PDA (5.4%), additional muscular VSD (5.4%) and coronary artery abnormalities (4.9%).
